# Multiplex connectomics reveal altered networks in frontotemporal dementia: A multisite study

**DOI:** 10.1162/netn_a_00448

**Published:** 2025-04-30

**Authors:** Sunil Kumar Khokhar, Manoj Kumar, Faheem Arshad, Sheetal Goyal, Megha Tiwari, Nithin Thanissery, Subasree Ramakrishnan, Chandana Nagaraj, Rajan Kashyap, Sandhya Mangalore, Tapan K. Gandhi, Suvarna Alladi, Rose Dawn Bharath, for the frontotemporal lobar degeneration neuroimaging initiative

**Affiliations:** Department of Neuroimaging and Interventional Radiology, National Institute of Mental Health and Neurosciences (NIMHANS), Bengaluru, India; Department of Neurology, NIMHANS, Bengaluru, India; Department of Electrical Engineering, Indian Institute of Technology (IIT) Delhi, New Delhi, India

**Keywords:** Multiplex, Connectomics, Graph theory, Frontotemporal dementia, Fluorodeoxyglucose-positron emission tomography, Cortical thickness

## Abstract

A network neuroscience perspective can significantly advance the understanding of neurodegenerative disorders, particularly frontotemporal dementia (FTD). This study employed an innovative multiplex connectomics approach, integrating cortical thickness (CTH) and fluorodeoxyglucose-positron emission tomography (FDG-PET) in a dual-layer model to investigate network alterations in FTD subtypes across two geographically distinct sites. The cohort included groups of behavioral variant FTD (bvFTD), primary progressive aphasia (PPA), mild cognitive impairment (MCI), and cognitively normal (CN) individuals who were analyzed from two separate sites. Site 1 included 28 bvFTD, 20 PPA, and 27 MCI participants, whereas Site 2 included 26 bvFTD, 43 PPA, and 43 CN individuals, respectively. Utilizing CTH and FDG-PET data after standard preprocessing, a multiplex network pipeline in BRAPH2 toolbox was used to derive multiplex participation coefficient (MPC) between the groups. The analysis revealed an increase in global MPC as an indicator of disease in PPA at both sites. Additionally, nodal MPC alterations in the anterior cingulate, frontal, and temporal lobes in PPA were compared with bvFTD. Comparisons with the CN showed that nodal MPC alterations were more extensive in PPA when compared with bvFTD. These findings underscore the potential utility of multiplex connectomes for identifying network disruptions in neurodegenerative disorders, offering promising implications for future research and clinical applications.

## Author Summary

This study uses multiplex connectomics, a method combining cortical thickness and glucose metabolism data, to investigate frontotemporal dementia (FTD) across two sites. Behavioral variant FTD (bvFTD) and primary progressive aphasia showed global and nodal brain network alterations. PPA exhibited more widespread disruptions in the anterior cingulate, frontal, and temporal lobes than bvFTD, suggesting greater network changes. bvFTD showed distinct alterations in the left caudal anterior cingulate and temporal pole compared with mild cognitive impairment and more extensive changes compared with cognitively normal individuals. These findings highlight the value of multiplex connectomics in identifying complex brain network changes in FTD and emphasize the need for further research to uncover the mechanisms behind these alterations.

## INTRODUCTION

The rapid growth of India’s aging population, coupled with a rise in modifiable risk factors—air pollution, hypertension, traumatic brain injury, and obesity—presents a significant challenge for the nation’s public health and social care systems (Livingston et al., 2020). Neurodegenerative disorder is a major cause of cognitive disability and dependency among the elderly and is expected to affect millions of Indian families over the next 2 decades (Ravindranath & Sundarakumar, 2021). Frontotemporal dementia (FTD) is an early-onset neurodegenerative disorder that primarily affects the frontal and temporal lobes of the brain leading to a range of behavioral and language impairments (Rascovsky et al., 2011; Warren et al., 2013). FTD group has a behavioral variant FTD (bvFTD) presenting with alterations in personality, cognition, and behavior, and a primary progressive aphasia (PPA) variant predominantly presenting with disorders in language and speech (Gorno-Tempini et al., 2011; Rascovsky et al., 2011). Survival differs across these clinical groups, with patients diagnosed with the semantic variant of PPA having an average survival of up to 12.2 years, while those with bvFTD have a survival of around 8.2 years (Kansal et al., 2016). Pathologically, FTD has the intracellular deposition of Tau, TAR DNA binding protein-43, or *fused in sarcoma* (RNA binding protein; Mackenzie et al., 2010) in the majority of patients. It is important to note the lack of correlation between pathological subtypes with clinical syndromes, wherein one clinical subtype could have several pathological subtypes and one pathological subtype could have varying clinical presentations.

Multilayer network approaches integrate structural and metabolic connectivity, providing a more comprehensive understanding of the brain’s network alterations than single-layer methods. Studies have shown that multilayer approaches can reveal hidden network properties related to disease progression and cognitive decline such as Alzheimer’s disease (AD) (Canal-Garcia et al., 2022). In the context of bvFTD and PPA, the added value lies in the potential to capture complex alterations that may not be detectable in single-layer metrics. These multilayer analyses can help in identifying specific biomarkers for differentiating these syndromes, particularly through the examination of region-specific nodal changes.

Neuroimaging is a key component in the diagnostic workup of patients with FTD and reveals characteristic anterior cingulate and fronto-insular hypometabolism or atrophy in bvFTD and asymmetric temporal prominent atrophy in the language variant of PPA supporting the clinical syndromes of FTD (Cerami et al., 2017; Gorno-Tempini et al., 2004; Rogalski et al., 2011). These regions are consistent with disease epicenters representing neurons with heightened vulnerability to disease especially in the early stages of the disease onset (Seeley et al., 2009). Existing research using whole-brain, data-driven, graph theory methods (Bullmore & Sporns, 2009) utilizing measures of segregation (clustering coefficient, local efficiency, modularity), integration (path-length, global efficiency, participation coefficient), and centrality (betweenness centrality) when compared with healthy controls have revealed significant anterior predominant alterations in frontal, temporal, and limbic regions in bvFTD (Nigro et al., 2021) and bilateral left more than right frontotemporal regions in PPA (Nigro et al., 2022; Tao et al., 2020) reiterating the epicenter concept of the disease. Several studies also have investigated network differences when comparing disease-matched controls. The comparison of AD with bvFTD has shown a higher participation coefficient in the left frontomedial cortices in AD compared with bvFTD patients (Ng et al., 2021). Resting-state functional MRI (rs-fMRI) studies have shown significant network alterations in the parietal lobe in early-onset AD compared with bvFTD (Filippi et al., 2017). Unilayer networks in neuroimaging refer to the analysis within a single layer of data, either from fluorodeoxyglucose-positron emission tomography (FDG-PET), rs-fMRI, or structural MRI, measuring regional variations of glucose metabolism, BOLD changes, or cortical thickness (CTH). A multilayer network, however, consists of several distinct unilayer networks, which use identical regions of interest (ROI) as nodes and with the concept of adjacency matrix (De Domenico, 2017), which measures edges between layers to identify regions with similar network measures across modalities (Battiston et al., 2014). Several network measures, like multiplex participation coefficient (MPC), multiplex clustering coefficient, and multiplex overlapping degree, can be derived from such networks (Canal-Garcia et al., 2022). Nodal MPC is an integration measure that can be used to detect highly connected hubs in both layers, and increasing nodal MPC indicates densely connected hubs that are similar in both layers. A higher MPC in multiplex terminology does not reflect increased hubness of the nodes; it reflects the differential contribution of the layers as the edges define the participation coefficient strictly between layers. Multiplex clustering coefficient is a segregation measure of the number of triangles formed by the nodes across layers, indicating their cliqueness. Multiplex overlapping degrees is another measure of segregation, which is the sum of the degree of the node in both layers. Few studies have used a multiplex model using rs-fMRI, electroencephalogram, or magnetoencephalography in dementia (Klepl et al., 2023; Wang et al., 2021). A recent study using CTH and amyloid PET using multiplex graph measures in AD with amyloid beta (Aβ+) patients found alterations in MPC in several regions including the medial temporal lobe, cuneus, and anterior cingulate regions (Canal-Garcia et al., 2022). Another study using multiple frequency bands in rs-fMRI study as different layers (Wang et al., 2021) revealed reduced MPC in AD compared with controls.

Given the varying clinical profile, imaging, and network features in patients with bvFTD and PPA, in the current study, we used the advanced imaging modalities in the clinical care of patients—CTH and FDG-PET—within a multiplex connectome to explore differences in the multilayer graph measures between bvFTD and PPA. Our study used mild cognitive impairment (MCI) to compare with dementia patients. We also used publicly available Neuroimaging in Frontotemporal Dementia (NIFD) data in patients with bvFTD, PPA, and cognitively normal (CN) to evaluate the consistency of the pipeline. We hypothesized that bvFTD will show significant differences in multiplex network measures compared with PPA based on the existing clinical and imaging evidence.

## METHODS

### Demographic Information of Participants

In this ambispective comparative cohort study of consecutive patients referred for positron emission tomography-magnetic resonance imaging between 2016 and 2022, we examined a total of 75 patients (from Site 1: 28 bvFTD, 20 PPA, and 27 MCI). The severity of dementia was assessed using the Clinical Dementia Rating (CDR) scale. Cognitive evaluations were conducted using Addenbrooke’s Cognitive Examination-III (ACE-III; Mekala et al., 2020). The diagnosis of FTD was made in patients using the standard criteria (Gorno-Tempini et al., 2011; Rascovsky et al., 2011), while the diagnosis of MCI was made based on the modified Petersen criteria (Petersen, 2004). In the retrospective group from Site 1 (31 subjects), CDR scales and ACE-III scores were extracted from the patient case files, and imaging data were obtained from the imaging data bank. For all the subjects in the prospective group (44 subjects), informed consent, and clinical and behavioral scores were obtained in person. Additionally, for retrospective cases, we received the approval from Institute Ethics Committee for waiver of consent. The study protocol was approved by this committee (Ethics NO. NIMH/DO/[BS&NS DIV.] 2019–20). All methods were carried out in compliance with the approved Indian Council of Medical Research guidelines. For the purpose of comparison with global data, we used the 112 patients from NIFD to replicate the pipeline (site 2, 26 bvFTD, 43 PPA, and 43 CN; https://adni.loni.usc.edu). Since ACE-III was unavailable for the Site 2 data, Mini-Mental State Examination (MMSE) scores were used for cognitive evaluation.

Comparing the groups, the MCI from Site 1 was comparable to bvFTD and PPA (Table 1) in age, gender, and disease duration. bvFTD and PPA revealed significant differences in ACE-III score compared with MCI and bvFTD had significantly lower years of education compared with MCI (bvFTD = 12.86 ± 3.99, MCI = 15.56 ± 3.10; Table 1). In Site 2, there were no significant differences in gender and years of education between these groups; however, the bvFTD was significantly younger than PPA and CN (bvFTD = 61.85 ± 6.56, PPA = 66.81 ± 8.04, and CN = 62.16 ± 7.66 years). MMSE scores exhibited no significant difference between bvFTD and PPA (Table 2). The effects of age, gender, and education were regressed from the analysis at the time of network construction, to ensure that the results were unaffected by these confounds.

**Table 1.** Demographic, clinical characteristics MCI, bvFTD, and PPA groups from Site 1

**Characteristic**		**MCI**		**FTD**				***F***	**PR(> F)**	**Post hoc (Bonferroni)**
				**bvFTD**		**PPA**					
***N***		27		28		20		--	--	--
**Gender (M:F)**		20:07		18:10		13:07		7.61	0.02	**NS**
		Mean	*SD*	Mean	*SD*	Mean	*SD*			
**Age (years)**		63.52	9.02	63.07	9.08	59.20	6.74	1.65	0.20	**NS**
**Education (years)**		15.56	3.05	12.86	3.99	14.65	3.99	3.67	0.03	**MCI > bvFTD**
**Duration of illness (years)**		2.24	1.89	3.02	1.40	3.10	1.17	2.33	0.10	**NS**
**ACE III**		81.13	11.41	58.04	13.92	39.55	21.84	39.68	<0.001	**MCI > bvFTD, MCI > PPA & bvFTD > PPA**
**CDR**	**Very mild**	27		05		05		23.38	<0.001	**PPA > MCI & bvFTD > MCI**
	**Mild**	--		15		09				
	**Moderate**	--		06		06				
	**Severe**	--		02		--				

**Table 2.** Demographic, clinical characteristics of CN, bvFTD, and PPA groups from Site 2

**Characteristic**		**CN**		**FTD**				***F***	**PR(> F)**	**Post hoc (Bonferroni)**
				**bvFTD**			**PPA**			
***N***		43		26		43		--	--	--
**Gender (M:F)**		17:26		17:09		24:19		4.79	0.09	**NS**
		Mean	*SD*	Mean	*SD*	Mean	*SD*			
**Age (years)**		62.16	7.66	61.85	6.56	66.81	8.04	5.13	0.01	**PPA > bvFTD & PPA > CN**
**Education (years)**		20.93	17.36	15.65	3.17	16.02	2.80	2.72	0.07	**NS**
**MMSE**		28.51	5.24	21.23	7.39	17.65	9.22	22.69	<0.001	**CN > bvFTD & CN > PPA**
**CDR**	**None**	43		--		--		92.91	<0.001	**bvFTD > CN, PPA > CN & bvFTD > PPA**
	**Very mild**	--		01		24				
	**Mild**	--		09		10				
	**Moderate**	--		16		09				
	**Severe**	--		--		--				

### Acquisition Parameters

#### Magnetization prepared rapid acquisition gradient-recalled echo (MPRAGE).

At Site 1, subjects underwent structural MRI on a 3 Tesla (Biograph mMR, Siemens, Erlangen, Germany) scanner using a 16-channel transmit receive head coil. Anatomic images were obtained using a three-dimensional T1-weighted MPRAGE sequence; additional conventional sequences, fluid-attenuated inversion recovery, T2, and susceptibility weighted imaging were acquired; and structural and unrelated metabolic abnormalities were excluded in all patients by domain experts (e.g., Dr. R.D.B., Dr. S.M., Dr. C.N.). At Site 2, subjects were imaged on a 3 Tesla scanner (Discovery MR 750, GE Medical Systems), where 3D T1-weighted MPRAGE sequences were also obtained. Detailed acquisition parameters are provided in Supporting Information Table S1.

#### FDG-PET.

Subjects were administered FDG 185 ± 10%, Megabecquerel (5.0 mCi) intravenously 30 min before acquiring PET scan in list mode for 15 min. Standard corrections for attenuation, scatter, random coincidences, decay, and a 5- mm Gaussian postreconstruction filter were applied. The acquisition parameters in Site 2 were identical, and a comparative parameter table is provided in Supporting Information Table S1. There were no differences in the acquisition parameters between the retrospective and prospective groups of patients. Since Site 2 had 30 min of PET data, only the first 15 min of data was used for analysis to make it comparable with Site 1 data in the current study.

### Preprocessing

#### CTH.

The process of reconstructing the cortex and determining CTH was carried out using the FreeSurfer image analysis tool version 6.05 (Fischl, 2012; https://surfer.nmr.mgh.harvard.edu). The pipeline within the FreeSurfer suite involves an automated process for removing the skull, transforming images into Talairach space, segmenting subcortical gray and white matter, and delineating the boundaries between gray and white matter. Subsequently, the cortical surface underwent reconstruction through precise intersubject alignment methods, resulting in a detailed measurement of CTH and categorization into various predefined cortical brain regions as per different atlases (Dale et al., 1999). CTH was determined as the minimum distance between the junction of gray and white matter and the pial surface. The results of these preliminary processing stages were visually examined to confirm that the analyses were conducted accurately. To ensure consistency with PET segmentation, the cortical surface was divided into unique areas based on gyral and sulcal structures using the Desikan-Killiany (DK) atlas, which identifies 34 regions in each hemisphere (Desikan et al., 2006).

#### FDG-PET.

PETSurfer analysis tool v6.05 was used for FDG-PET image analysis (Greve et al., 2014). PETSurfer performs PET processing by utilizing the result from FreeSurfer, involving registration into the Montreal Neurological Institute space. Further, it provides partial volume correction using geometric transfer matrix methods for the partial volume effect, which can impact the accuracy of PET measurements (Greve et al., 2016). After segmentation and registration, the pipeline applies PET Surfer and calculates the standardized uptake value ratio (SUVR) values with pons as a reference region, volume size in the region, and voxel variance. The DK atlas was used to parcellate the cortical surface into 34 regions per hemisphere (Desikan et al., 2006; Khokhar et al., 2023). Additionally, we also utilized the Destrieux atlas, which comprises 74 regions per hemisphere, to assess the stability of our findings and to explore whether consistent results could be obtained using two different atlases. The results based on the Destrieux atlas are included in the supplementary material for reference. Further, we quantified the metabolic index for FDG-PET using the approach described by Thibaut et al. (2021) to generate a mean SUV image representing FDG uptake (Supporting Information Figure S3).

### Multilayer Graph Theory

For the analysis of multiplex data (derived from structural MRI and FDG-PET), we used the BRAPH2 toolbox v2.03, specifically designed for a multilayer graph analysis. The multilayer graph method involved the creation of a multiplex network with two specific layers: the CTH and the FDG-PET network. Nodes within each network represented distinct ROIs. In the CTH network, these nodes were based on the average CTH of 68 different brain regions. Additionally, we extended our analysis by incorporating the Destrieux atlas, which includes 74 regions in each hemisphere. For the FDG-PET network, the same brain regions were used, but the nodes were measured by the mean SUVR values. We conducted separate linear regressions for each ROI, employing age, sex, and education as independent predictors, and either CTH or FDG-PET measure as the dependent variable.

$$Y_{i}=\beta_{0}+\beta_{1}(\text{Age})+\beta_{2}(\text{Sex})+\beta_{3}(\text{Education})+\varepsilon_{i}$$

where *Y*_*i*_ represents the dependent variable (CTH or FDG-PET) for ROI*_i_*; *β*_0_ is the intercept; *β*_1_, *β*_2_, and *β*_3_ are the coefficients for age, sex, and education, respectively; *ϵ_i_* represents the error term for ROI*_i_*. Subsequently, the residuals derived from these regressions were utilized as input for constructing the networks.

**Figure 1.**
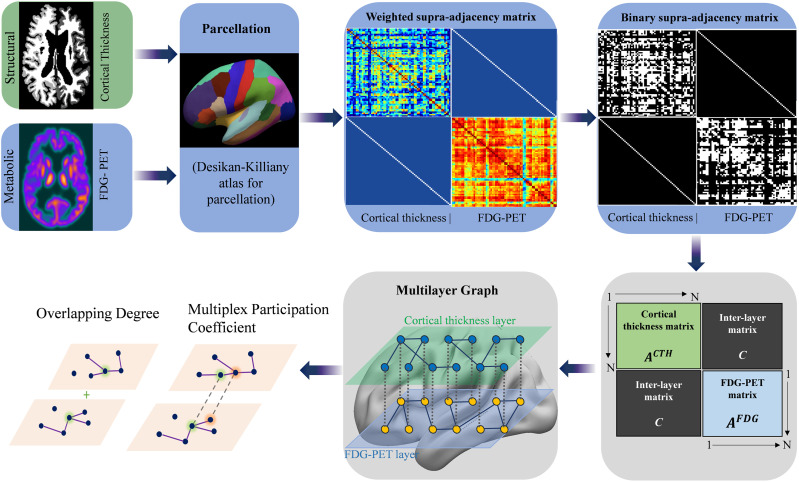
Schematic representation of the image processing and multilayer graph theory analysis pipeline. CTH and FDG-PET values are extracted for each ROI. Pearson’s correlation coefficients are calculated between all ROI pairs within each group, generating group-level connectivity matrices for each modality (CTH and FDG-PET). These intralayer matrices are then integrated into a supra-adjacency matrix to facilitate multiplex graph analysis. (*N* represents number of nodes in matrix.)

The network construction procedure involves the following steps:

1. CTH and FDG-PET values are obtained for each ROI for each participant. 2. Pearson’s correlation coefficients are calculated between all pairs of ROIs within each group, resulting in a group-level connectivity matrix for each modality (CTH and FDG-PET). 3. The intralayer matrices are then combined into a supra-adjacency matrix for the multiplex analysis. This supra-adjacency matrix represents a multiplex network that integrates the intralayer connectivity matrices for CTH and FDG-PET. The main diagonal of the matrix represents intralayer connections, while the off-diagonal elements represent interlayer connections. These layers were utilized to form a multiplex network using binary undirected graphs from a weighted supra-adjacency matrix W derived at fixed thresholds (Figure 1). The multiplex network is represented through a weighted supra-adjacency matrix, denoted as W (Canal-Garcia et al., 2022). This matrix consists of intralayer adjacency matrices aligned along main diagonal and interlayer connections in off-diagonal blocks:

$$W=\left[\begin{array}{cc} A^{CTH} & C\\ C & A^{FDG} \end{array}\right]$$

where *A^CTH^* is the weighted adjacency matrix for CTH, *A^FDG^* is the weighted adjacency matrix for FDG-PET, and *C* represents interlayer adjacency matrices. In this configuration, the intralayer adjacency matrices are positioned on the main diagonal, and the interlayer adjacency matrices, which connect corresponding nodes across different layers, are located in the off-diagonal blocks (De Domenico et al., 2013). The analyses were conducted across a range of thresholds from 0.4 to 0.7 in increments of 0.05, or 40% to 70% in the steps of 5% (Sanabria-Diaz et al., 2010). In addition to network results for multiple comparisons, a false discovery rate (FDR) procedure was applied. Various parameters, as described below, were derived from the multiplex network.

#### Overlapping degree (global).

The average overlapping degree of a graph is calculated by taking the mean of the total degrees of a node, summed across all layers in the network.

$$o_{ij}=\sum_{\alpha }k_{i}^{\alpha }$$

where the degree of node *i* in layer *α* as $k_{i}^{\alpha }$ = *∑_j_* $a_{ij}^{\alpha }$ (Boccaletti et al., 2014).

#### MPC (nodal).

The integration between layers are quantified using the nodal MPC. The nodal MPC, *p*_*i*_, measures how uniformly a node *i* is connected across the layers of the multilayer network: Nodes with higher values of pi have a similar participation coefficient across the different network layers of the multiplex network (Battiston et al., 2014). The MPC of node *i* is defined as

$$p_{i}=\frac{M}{M-1}\left[1-\sum_{a=1}^{M}{\left(\frac{k_{i}^{\left[\alpha \right]}}{o_{i}}\right)}^{2}\right]$$

where M is the number of layers, $k_{i}^{\left[\alpha \right]}\;$is the degree in the *α*-th layer, and *o*_*i*_ = $\sum_{\alpha }k_{i}^{\left[\alpha \right]}$ is the overlapping degree of node *i*.

#### MPC (global).

Global MPC represents the mean value of the nodal participation coefficients across the various layers of a network.

### Statistical Analysis

To assess differences between groups in demographic variables, statistical analysis was performed with “*scipy*” library in Python. Differences between groups in age, education, and ACE-III/MMSE were tested using an analysis of variance for continuous variables, while gender differences between groups were tested using a chi-square test. Post hoc analyses using Bonferroni-corrected pairwise *t* tests, following a Kruskal–Wallis test for the ordinal variable CDR, were conducted into pairwise group differences with a significance level of 0.05.

For multiplex analysis, we carried out a nonparametric permutation test with 5,000 permutations. Group differences were considered significant for a two-tailed *p* value based on 95% confidence intervals. FDR corrections (Benjamini & Hochberg, 1995) were applied on multiplex graph measures to control for multiple comparisons (*p* < 0.05). The multiplex analysis results were visualized with “*fsbrain*” library, a visualization for brain statistics R-package (Schäfer & Ecker, 2020) using the DK atlas.

## RESULTS

In this multisite analysis of multiplex network from sMRI and FDG-PET, PPA revealed significantly increased global MPC when compared with MCI and CN in Site 1 and Site 2, respectively. Nodal MPC revealed more widespread alterations when compared with controls with significant alterations in anterior cingulate and temporal lobes in PPA when compared with bvFTD in both Site 1 and Site 2.

### Multiplex Global Measures

#### Overlapping degree average (global).

There was a significantly reduced global overlapping degree in patients with PPA when compared with CN in Site 2 (*d* = 0.7, diff = 0.13). However, when comparing bvFTD to MCI, bvFTD to CN, PPA to MCI, and bvFTD to PPA (Sites 1 & 2), no significant differences were observed in the overlapping degree.

#### MPC (global).

There was an increase in the global MPC in patients with bvFTD when compared with PPA in Site 2 (*d* = 0.4, 0.45, 0.5, 0.55, diff = −0.20, −0.24, −0.25, −0.27). PPA, when compared with MCI in Site 1 (*d* = 0.6, 0.65, 0.7, diff = 0.44, 0.47, 0.40), showed increase global MPC, also seen in bvFTD and PPA when compared with CN (*d* = 0.4, 0.45, 0.5, 0.55, 0.6, 0.65, 0.7; diff = 0.22, 0.26, 0.32, 0.38, 0.45, 0.65, 0.7 and *d* = 0.7; diff = 0.23, respectively). When comparing bvFTD to MCI, and bvFTD to PPA in Site 1, no significant differences were observed in the global MPC.

### MPC (Nodal).

Nodal MPC identified several alterations in the frontal and temporal lobes in both sites.

#### bvFTD versus PPA.

**Figure 2.**
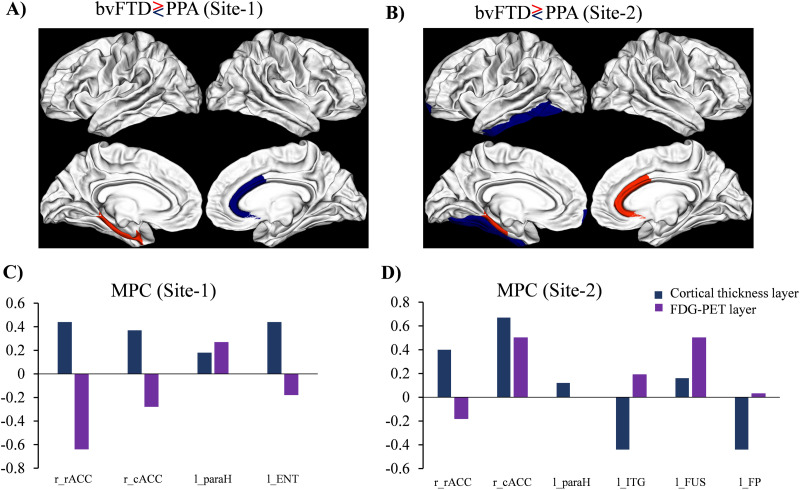
Nodal MPC of significant differences between the bvFTD and PPA from Site 1 (A), and bvFTD versus PPA from Site 2 (B), two layers of connectivity imbalance can be seen for Site 1 and Site 2 (C and D, respectively) between bvFTD and PPA (FDR corrected at *p* < 0.05).

In Site 1, bvFTD showed increased MPC in the left entorhinal and parahippocampal regions compared with PPA, while the right rostral and caudal anterior cingulate regions had lower MPC in bvFTD compared with PPA (Figure 2A). In Site 2, the left parahippocampal region, similar to Site 1, and the *right*-rostral and caudal anterior cingulate regions exhibited increased MPC in bvFTD compared with PPA (Figure 2B). However, the *left-*inferior temporal, fusiform, and frontal pole regions showed lower MPC in bvFTD compared with PPA. As shown in Figures 2C and 2D, these connectivity differences indicate relative variations in the contribution of different layers between the groups. Figures 2C and 2D, where the increased nodal MPC in bvFTD in the left parahippocampal gyrus was mediated by the increased contribution of the FDG in Site 1 and CTH in Site 2, compared to PPA. When it comes to the right rostral and caudal anterior cingulate, it can be seen that the decreased MPC in Site 1 is mediated by the prominent contribution of the FDG layer and the increased MPC in Site 2 is due to the increased contribution of the CTH layer.

#### PPA versus MCI/CN.

**Figure 3.**
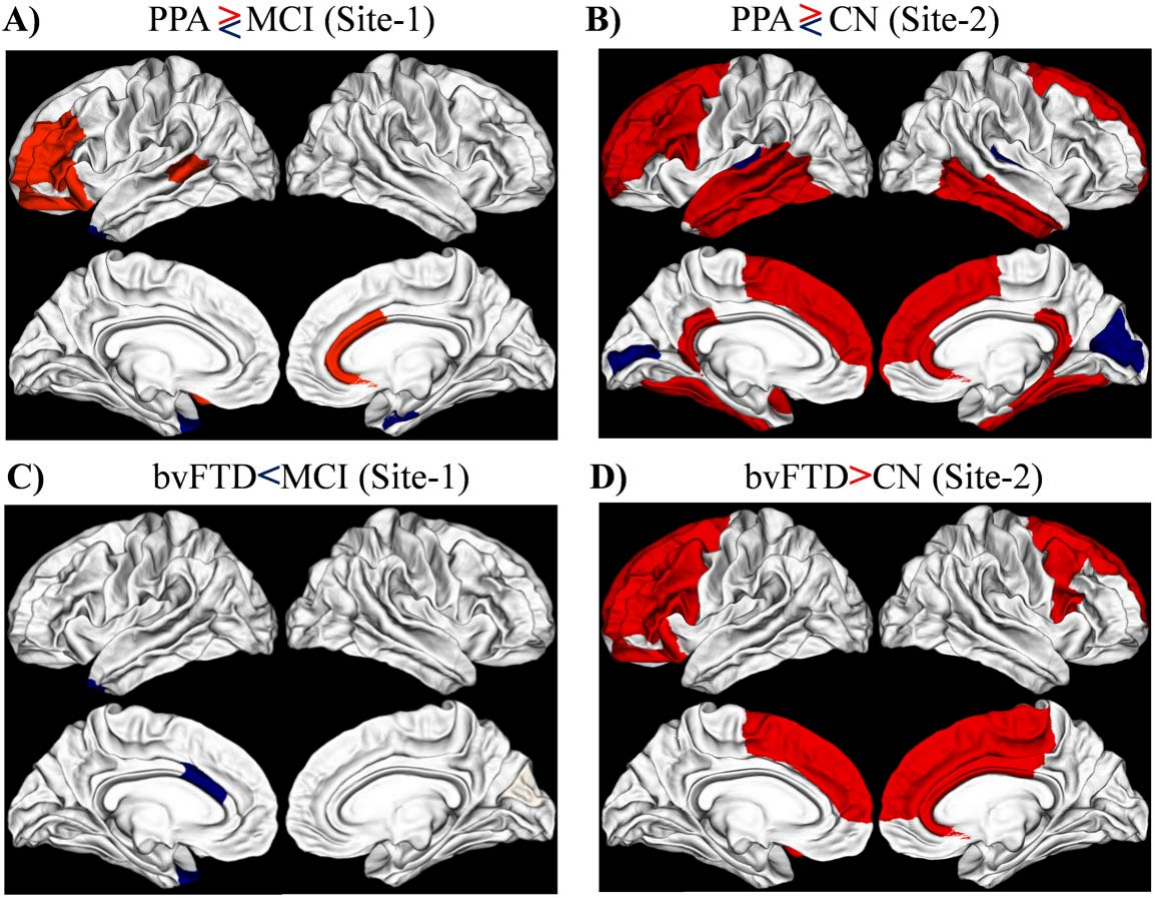
Nodal MPC shows the significant differences between the group PPA and MCI (A), PPA and CN (B), bvFTD and MCI (C), and bvFTD and CN (D) (FDR corrected at *p* < 0.05).

PPA revealed wider areas of altered MPC than bvFTD when compared with MCI/CN. Right-rostral anterior cingulate, caudal anterior cingulate, left lateral orbitofrontal, pars triangularis, rostral middle frontal, and banks of the superior temporal sulcus showed increased MPC in PPA when compared with MCI. The right entorhinal and left temporal pole showed decreased MPC in PPA when compared with MCI. In Site 2 data, PPA revealed increased nodal MPC in *right*-rostral anterior cingulate, parahippocampal, isthmus cingulate, inferior temporal, middle temporal, fusiform, frontal pole, superior frontal*, left*-inferior temporal, isthmus cingulate, middle temporal, pars opercularis, rostral middle frontal, caudal middle frontal, superior temporal, fusiform, left banks of the superior temporal sulcus, and frontal pole, superior frontal. Conversely, there were regions, the *right*—cuneus, pericalcarine, transverse temporal; ***left—***pericalcarine and transverse temporal, that revealed decreased MPC in PPA (Figures 3A and 3B).

#### bvFTD versus MCI/CN.

The left caudal anterior cingulate and temporal pole showed significantly lower MPC in bvFTD in comparison with MCI in Site 1. Increased MPC was more diffuse and bilateral involving the frontal lobes and cingulate gyrus when compared with healthy controls (site 2) than MCI (site 1). Specifically in Site 2, the *right*-posterior cingulate, rostral anterior cingulate, caudal anterior cingulate, pars opercularis, caudal middle frontal, paracentral, superior frontal, ***left-***lateral orbitofrontal, pars opercularis, pars triangularis, rostral middle frontal, caudal middle frontal, and superior frontal showed significantly higher MPC in bvFTD compared with CN (Figures 3C and 3D).

## DISCUSSION

The complex nature of neurodegenerative disorders necessitates a connectomics approach to understand their pathobiology *in vivo*, and the current study with multiplex global and nodal connectomics across two geographically distinct sites has revealed an increasing global network MPC as a marker of the disease in patients with PPA in both the sites. Nodal MPC using data-driven methods consistently identified alterations of several frontal and temporal lobe regions in both sites, supporting the clinical and pathological concept of disease epicenters. Both PPA and bvFTD show increased global and nodal MPC in similar frontal regions when compared with CN individuals, suggesting a shared network-level disruption in frontal circuits across these disorders. However, PPA showed distinct increases in nodal MPC within temporal regions, distinguishing it from MCI and highlighting the diagnostic potential of temporal network alterations, likely reflecting compensatory network changes associated with disease progression. Additionally, differences in nodal MPC in parahippocampal regions emerged as a critical feature for differentiating bvFTD from PPA.

Only a few studies on neurodegenerative disorders, such as AD, have investigated multilayer connectomes (Canal-Garcia et al., 2022; Wang et al., 2021) and have found decreased nodal participation coefficient in several regions in the medial temporal, anterior cingulate, and cuneus in patients with AD A*β*+ compared with CN A*β*− participants due to differential contributions from amyloid PET and CTH. However, it needs to be noted that the increase in global MPC found in this study does not have the same biological meaning as the increased participation coefficient found in unilayer studies (Ng et al., 2021; Tao et al., 2021). Despite not having the biological correlate, multiplex connectomics is useful as it identifies areas that are involved across both layers providing summative evidence from multiple modalities.

bvFTD revealed significant alterations in the left caudal anterior cingulate and temporal pole when compared with MCI and revealed more widespread alterations involving various parts of the right cingulate gyrus, right paracentral, bilateral superior and middle frontal gyrus, pars opercularis and left pars triangularis and lateral orbitofrontal region when compared with CN. It is important to note that the data-driven method using multilayer connectomics has identified these areas that have been already identified in the literature using unilayer connectomics (Agosta et al., 2013; Mandelli et al., 2018; Reyes et al., 2018; Sedeño et al., 2017), pathology, and clinical methods.

It is interesting to note that PPA revealed more extensive alterations compared with bvFTD in both sites involving the right anterior cingulate and left-lateral orbitofrontal, pars triangularis, rostral middle frontal, and banks of the superior temporal sulcus when compared with the MCI group and involving several regions of bilateral cingulate isthmus, middle and inferior temporal, fusiform, superior frontal and frontal poles, left middle and inferior frontal, pars opercularis, transverse temporal, and pericalcarine regions. Asymmetric left predominant involvement was noted in the left middle and inferior frontal, pars opercularis, frontal pole, and superior temporal sulcus.

Contrasting the bvFTD and PPA with CN revealed more areas with alterations than with MCI. It is logical to consider these differences to be due to varying disease stages spotlighting the longitudinal evolution of the disease with more areas involved as the disease progresses, which is amplified when compared with CN and minimized in comparison with the MCI. Notably, the asymmetric left prominent involvement of the frontal and temporal lobes in PPA in both sites is also interesting. Asymmetric involvement of the left frontal and temporal lobes is one of the imaging markers for patients with PPA with left anterioinferior temporal lobe in semantic variant PPA and left side inferior frontal and insula in progressive nonfluent aphasia (Cerami et al., 2017; Gorno-Tempini et al., 2004; Rogalski et al., 2011). Though the regions involved closely map the existing literature in FTD, it is intriguing to note widespread alterations in PPA compared with bvFTD in both sites. This is counterintuitive since clinically PPA is a milder form of disease with a better prognosis than bvFTD. FDG-PET and conventional MRI also reveal more focal disease in PPA as compared with bvFTD. One explanation for this result could be that the network changes denote both disease-induced changes and compensatory network modulations due to the disease (Liu et al., 2022). Our analysis of bvFTD versus PPA revealed significant differences in the right cingulate gyrus and the left parahippocampal gyrus in both sites. Additional involvement of the left entorhinal, fusiform gyrus, and frontal pole was identified in Site 2. Additional involvement of the left temporal and frontal areas in Site 2 can be partially explained by the differences in the severity of the diseases. CDR scores from Site 2 revealed a higher number of PPA patients to belong to the minor group whereas the majority of bvFTD belonged to the severe group. The CDR scores across groups were more balanced in Site 1. Though it is logical to presume that the differences are reflective of the contributions by PPA, a detailed unilayer network analysis will be required to further understand the biological meaning of this finding as the multiplex methodology depict contributions of the layers and not the disease. Since the derived regions indicate group results, it is difficult to correlate these with clinical and behavioral scores.

Multiplex connectomics provides a platform for the integration of multimodal techniques. Several studies using unilayer CTH and FDG-PET networks have revealed highly heterogeneous results with the involvement of several areas in the literature (Malpetti et al., 2019; Vijverberg et al., 2017). Some studies have also observed heterogeneity between FDG-PET and CTH patterns in FTD patients (Bejanin et al., 2020; Cerami et al., 2016). Some studies reveal increased connectivity, and some others reveal decreased connectivity when compared with CN, justified by differences in the temporal evolution of the disease, and varying image processing pipelines and statistical thresholds used while deriving the results. Studies from geographically different locations also add to the variability necessitating large data samples to comprehend disease pathobiology. It is important to note that the current multiplex connectomics study across two geographically different sites revealed increased global MPC in FTD with altered nodal MPC in several regions involving the frontal and temporal lobes. Though it is difficult to derive the network pathobiology in FTD from this data, since the results provided consistent results across two distinct data sets, multiplex connectomics by its very characteristic of defining edges between layers appears to be an appropriate tool in addressing the heterogeneity in data sets.

Despite the substantial contributions of our study in comprehending network changes in dementia through the innovative application of a multiplex network approach, certain limitations need acknowledgment. First, in the current study, the DK atlas was utilized for the parcellation of CTH and FDG uptake regions. A significant limitation stems from this atlas’s inability to delineate the thickness of subcortical brain regions and the cerebellum. As a result, this confines our understanding of the whole brain’s structural and metabolic interactions to a certain extent. Further, we did an analysis using Destrieux atlas and the findings showed concordance with DK atlas, and we found increasing global MPC in patients with PPA, when compared with MCI and CN in Site 1 and Site 2, respectively, and in bvFTD when compared with CN in and Site 2 with no differences between bvFTD and MCI in site 1. Nodal MPC also revealed significant alterations in bvFTD and PPA when compared with CN and MCI in the frontotemporal region aligning with the DK atlas; however, in bvFTD, when compared with PPA in both Site 1 and Site 2, more regions in the neocortical frontal, occipital, temporal and cingulate, which unlike in DK atlas, were limited to cingulate and temporal regions. These differences could be attributed to increased granularity of the Destrieux Atlas with 148 ROI, underscoring the importance of parcellation in the nodal participation coefficient (supporting information results).

Second, extracting graph measures from structural and PET data on an individual basis proved challenging, preventing correlation with clinical and cognitive scores. Recent developments in FDG-PET time series (functional PET) acquisition, combined with MR-PET hybrid scanners, could enhance this approach at the subject level. Third, the absence of healthy control subjects at Site 1 posed a significant limitation, potentially introducing biases. Fourth, the analytical framework primarily hinged on cross-sectional imaging data for executing multiplex analyses, which inherently restricted the investigation of temporal variations of FDG-PET and gray matter atrophy across groups. Fifth, our findings are limited by a smaller sample size, which may affect their generalizability. Additionally, we have included both the semantic variant and the nonfluent variant of PPA under the PPA category due to smaller numbers. The biological meaning of the network alterations could not be derived because of the interlayer measures employed, thereby limiting the interpretability of the clinical context. Subsequent research should incorporate other imaging techniques, like fMRI, functional FDG-PET and Tau-PET or Translocator Protein-PET, into multiplex analyses to gain insights into the relationship between brain atrophy, pathology, and other pathological processes in FTD. Moreover, longitudinal study designs could be instrumental in exploring the temporal trajectory of tau pathologies vis-a-vis gray matter atrophy. If the pipeline in the future can, in addition, provide within-network measures, it will be more useful in deriving clinical correlates.

### Conclusion

The current study using a multiplex connectomics approach revealed widespread changes in the MPC, in FTD subtypes consistently across geographically distinct sites using the most popular imaging tools—CTH and FDG-PET. These findings offer new insights into the interplay between metabolism and brain atrophy over the course of FTD and subtypes and move beyond traditional graph theory analyses based on unilayer brain networks.

## Acknowledgments

We would like to acknowledge the participants who took part in this study and the staff at the PET-MRI facility for their assistance in data acquisition. We would like to express our gratitude to the National Institute of Mental Health and Neuro Sciences (NIMHANS) for providing the resources.

Frontotemporal Lobar Degeneration Neuroimaging Initiative (FTLDNI) was funded through the National Institute of Aging and started in 2010. The primary goals of FTLDNI were to identify neuroimaging modalities and methods of analysis for tracking frontotemporal lobar degeneration and to assess the value of imaging versus other biomarkers in diagnostic roles. The Principal Investigator of NIFD was Dr. Howard Rosen, MD at the University of California, San Francisco. The data are the result of collaborative efforts at three sites in North America. For up-to-date information on participation and protocol, please visit https://memory.ucsf.edu/research/studies/nifd. Grant Number: R01 AG032306.

## Supporting Information

Supporting information for this article is available at https://doi.org/10.1162/netn_a_00448.

## Author Contributions

Sunil Kumar Khokhar: Conceptualization; Data curation; Formal analysis; Methodology; Resources; Software; Visualization; Writing – original draft; Writing – review & editing. Manoj Kumar: Investigation; Supervision; Validation; Writing – review & editing. Faheem Arshad: Data curation; Investigation; Resources; Validation; Writing – review & editing. Sheetal Goyal: Data curation; Investigation; Validation. Megha Tiwari: Data curation; Investigation; Methodology. Nithin Thanissery: Data curation; Methodology; Writing – review & editing. Subasree Ramakrishnan: Data curation; Supervision. Chandana Nagaraj: Supervision; Validation. Rajan Kashyap: Formal analysis; Methodology; Validation. Sandhya Mangalore: Methodology; Resources; Writing – review & editing. Tapan K. Gandhi: Conceptualization; Methodology; Supervision; Validation; Writing – review & editing. Suvarna Alladi: Conceptualization; Data curation; Supervision; Validation; Writing – review & editing. Rose Dawn Bharath: Conceptualization; Investigation; Methodology; Resources; Supervision; Validation; Writing – review & editing.

## Funding Statement

This research received no specific grant from any funding agency in the public, commercial, or not-for-profit sectors. The authors independently conducted the study, and all necessary resources and materials were provided by National Institute of Mental Health and Neuro Sciences.

## Author Disclosure Statement

The authors of this manuscript declare that they have no conflicts of interest to disclose concerning the contents of this research paper.

## Data Availability

• Data from Site 1 analyzed during the current study are not publicly available due to conditions of our ethics approval, particularly regarding patient confidentiality, and do not permit public archiving of the original data but are available from the corresponding author on reasonable request.

• Data from Site 2 analyzed during the current study are available in the *Frontotemporal Lobar Degeneration Neuroimaging Initiative* database repository, available at https://adni.loni.usc.edu.

## Author List of FTLDNI/NIFD Consortium

Howard Rosen; University of California, San Francisco (PI). Bradford C. Dickerson; Harvard Medical School and Massachusetts General Hospital. Kimoko Domoto-Reilly; University of Washington School of Medicine. David Knopman; Mayo Clinic, Rochester. Bradley F. Boeve; Mayo Clinic Rochester. Adam L. Boxer; University of California, San Francisco. John Kornak; University of California, San Francisco. Bruce L. Miller; University of California, San Francisco. William W. Seeley; University of California, San Francisco. Maria-Luisa Gorno-Tempini; University of California, San Francisco. Scott McGinnis; University of California, San Francisco. Maria Luisa Mandelli; University of California, San Francisco.

## Supplementary Material



## TECHNICAL TERMS

Multiplex connectomics: An approach that integrates multiple types of data (such as CTH and FDG-PET) into a unified network model to analyze brain connectivity patterns across different modalities.
Multiplex network: A multiplex network is a specific type of multilayer network where interlayer connections exist solely between the corresponding nodes across different layers.
Multiplex participation coefficient (MPC): A measure in multiplex network analysis that quantifies the extent to which a node (ROI) participates in different layers (modalities) of the network.
Overlapping degree: The overlapping degree of a graph is the sum of the degrees of a node in all layers.
Supra-adjacency matrix: A matrix representing the connections in a multiplex network, containing intralayer adjacency matrices on the diagonal and interlayer adjacency matrices on the off-diagonal.

## References

[bib1] Agosta, F., Sala, S., Valsasina, P., Meani, A., Canu, E., Magnani, G., … Filippi, M. (2013). Brain network connectivity assessed using graph theory in frontotemporal dementia. Neurology, 81(2), 134–143. 10.1212/WNL.0b013e31829a33f8, 23719145

[bib2] Battiston, F., Nicosia, V., & Latora, V. (2014). Structural measures for multiplex networks. Physical Review E, 89(3), 032804. 10.1103/PhysRevE.89.032804, 24730896

[bib3] Bejanin, A., Tammewar, G., Marx, G., Cobigo, Y., Iaccarino, L., Kornak, J., … Rabinovici, G. D. (2020). Longitudinal structural and metabolic changes in frontotemporal dementia. Neurology, 95(2), e140–e154. 10.1212/WNL.0000000000009760, 32591470 PMC7455324

[bib4] Benjamini, Y., & Hochberg, Y. (1995). Controlling the false discovery rate: A practical and powerful approach to multiple testing. Journal of the Royal Statistical Society: Series B (Methodological), 57(1), 289–300. 10.1111/j.2517-6161.1995.tb02031.x

[bib5] Boccaletti, S., Bianconi, G., Criado, R., Del Genio, C. I., Gómez-Gardeñes, J., Romance, M., … Zanin, M. (2014). The structure and dynamics of multilayer networks. Physics Reports, 544(1), 1–122. 10.1016/j.physrep.2014.07.001, 32834429 PMC7332224

[bib6] Bullmore, E., & Sporns, O. (2009). Complex brain networks: Graph theoretical analysis of structural and functional systems. Nature Reviews Neuroscience, 10(3), 186–198. 10.1038/nrn2575, 19190637

[bib7] Canal-Garcia, A., Gómez-Ruiz, E., Mijalkov, M., Chang, Y.-W., Volpe, G., Pereira, J. B., & Alzheimer’s Disease Neuroimaging Initiative. (2022). Multiplex connectome changes across the Alzheimer’s disease spectrum using gray matter and amyloid data. Cerebral Cortex, 32(16), 3501–3515. 10.1093/cercor/bhab429, 35059722 PMC9376877

[bib8] Cerami, C., Dodich, A., Greco, L., Iannaccone, S., Magnani, G., Marcone, A., … Perani, D. (2017). The role of single-subject brain metabolic patterns in the early differential diagnosis of primary progressive aphasias and in prediction of progression to dementia. Journal of Alzheimer’s Disease, 55(1), 183–197. 10.3233/JAD-160682, 27662315 PMC5115609

[bib9] Cerami, C., Dodich, A., Lettieri, G., Iannaccone, S., Magnani, G., Marcone, A., … Perani, D. (2016). Different FDG-PET metabolic patterns at single-subject level in the behavioral variant of fronto-temporal dementia. Cortex, 83, 101–112. 10.1016/j.cortex.2016.07.008, 27498041

[bib10] Dale, A. M., Fischl, B., & Sereno, M. I. (1999). Cortical surface-based analysis. I. Segmentation and surface reconstruction. NeuroImage, 9(2), 179–194. 10.1006/nimg.1998.0395, 9931268

[bib11] De Domenico, M. (2017). Multilayer modeling and analysis of human brain networks. GigaScience, 6(5), 1–8. 10.1093/gigascience/gix004, 28327916 PMC5437946

[bib12] De Domenico, M., Solé-Ribalta, A., Cozzo, E., Kivelä, M., Moreno, Y., Porter, M. A., … Arenas, A. (2013). Mathematical formulation of multilayer networks. Physical Review X, 3(4), 041022. 10.1103/PhysRevX.3.041022

[bib13] Desikan, R. S., Ségonne, F., Fischl, B., Quinn, B. T., Dickerson, B. C., Blacker, D., … Killiany, R. J. (2006). An automated labeling system for subdividing the human cerebral cortex on MRI scans into gyral based regions of interest. NeuroImage, 31(3), 968–980. 10.1016/j.neuroimage.2006.01.021, 16530430

[bib14] Filippi, M., Basaia, S., Canu, E., Imperiale, F., Meani, A., Caso, F., … Agosta, F. (2017). Brain network connectivity differs in early-onset neurodegenerative dementia. Neurology, 89(17), 1764–1772. 10.1212/WNL.0000000000004577, 28954876 PMC5664301

[bib15] Fischl, B. (2012). FreeSurfer. NeuroImage, 62(2), 774–781. 10.1016/j.neuroimage.2012.01.021, 22248573 PMC3685476

[bib16] Gorno-Tempini, M. L., Dronkers, N. F., Rankin, K. P., Ogar, J. M., Phengrasamy, L., Rosen, H. J., … Miller, B. L. (2004). Cognition and anatomy in three variants of primary progressive aphasia. Annals of Neurology, 55(3), 335–346. 10.1002/ana.10825, 14991811 PMC2362399

[bib17] Gorno-Tempini, M. L., Hillis, A. E., Weintraub, S., Kertesz, A., Mendez, M., Cappa, S. F., … Grossman, M. (2011). Classification of primary progressive aphasia and its variants. Neurology, 76(11), 1006–1014. 10.1212/WNL.0b013e31821103e6, 21325651 PMC3059138

[bib18] Greve, D. N., Salat, D. H., Bowen, S. L., Izquierdo-Garcia, D., Schultz, A. P., Catana, C., … Johnson, K. A. (2016). Different partial volume correction methods lead to different conclusions: An (18)F-FDG-PET study of aging. NeuroImage, 132, 334–343. 10.1016/j.neuroimage.2016.02.042, 26915497 PMC4851886

[bib19] Greve, D. N., Svarer, C., Fisher, P. M., Feng, L., Hansen, A. E., Baare, W., … Knudsen, G. M. (2014). Cortical surface-based analysis reduces bias and variance in kinetic modeling of brain PET data. NeuroImage, 92, 225–236. 10.1016/j.neuroimage.2013.12.021, 24361666 PMC4008670

[bib20] Kansal, K., Mareddy, M., Sloane, K. L., Minc, A. A., Rabins, P. V., McGready, J. B., & Onyike, C. U. (2016). Survival in frontotemporal dementia phenotypes: A meta-analysis. Dementia and Geriatric Cognitive Disorders, 41(1–2), 109–122. 10.1159/000443205, 26854827

[bib21] Khokhar, S. K., Kumar, M., Kumar, S., Manae, T., Thanissery, N., Ramakrishnan, S., … Bharath, R. D. (2023). Alzheimer’s disease is associated with increased network assortativity: Evidence from metabolic connectivity. Brain Connectivity, 13(10), 610–620. 10.1089/brain.2023.0024, 37930734

[bib22] Klepl, D., He, F., Wu, M., Blackburn, D. J., & Sarrigiannis, P. G. (2023). Cross-frequency multilayer network analysis with bispectrum-based functional connectivity: A study of Alzheimer’s disease. Neuroscience, 521, 77–88. 10.1016/j.neuroscience.2023.04.008, 37121381

[bib23] Liu, L., Chu, M., Nie, B., Liu, L., Xie, K., Cui, Y., … Wu, L. (2022). Reconfigured metabolism brain network in asymptomatic microtubule-associated protein tau mutation carriers: A graph theoretical analysis. Alzheimer’s Research & Therapy, 14(1), 52. 10.1186/s13195-022-01000-z, 35410286 PMC8996677

[bib24] Livingston, G., Huntley, J., Sommerlad, A., Ames, D., Ballard, C., Banerjee, S., … Mukadam, N. (2020). Dementia prevention, intervention, and care: 2020 report of the Lancet Commission. Lancet, 396(10248), 413–446. 10.1016/S0140-6736(20)30367-6, 32738937 PMC7392084

[bib25] Mackenzie, I. R. A., Neumann, M., Bigio, E. H., Cairns, N. J., Alafuzoff, I., Kril, J., … Mann, D. M. A. (2010). Nomenclature and nosology for neuropathologic subtypes of frontotemporal lobar degeneration: An update. Acta Neuropathologica, 119(1), 1–4. 10.1007/s00401-009-0612-2, 19924424 PMC2799633

[bib26] Malpetti, M., Carli, G., Sala, A., Cerami, C., Marcone, A., Iannaccone, S., … Perani, D. (2019). Variant-specific vulnerability in metabolic connectivity and resting-state networks in behavioural variant of frontotemporal dementia. Cortex, 120, 483–497. 10.1016/j.cortex.2019.07.018, 31493687

[bib27] Mandelli, M. L., Welch, A. E., Vilaplana, E., Watson, C., Battistella, G., Brown, J. A., … Gorno-Tempini, M. L. (2018). Altered topology of the functional speech production network in non-fluent/agrammatic variant of PPA. Cortex, 108, 252–264. 10.1016/j.cortex.2018.08.002, 30292076 PMC6317366

[bib28] Mekala, S., Paplikar, A., Mioshi, E., Kaul, S., Divyaraj, G., Coughlan, G., … Alladi, S. (2020). Dementia diagnosis in seven languages: The Addenbrooke’s Cognitive Examination-III in India. Archives of Clinical Neuropsychology, 35(5), 528–538. 10.1093/arclin/acaa013, 32188967

[bib29] Ng, A. S. L., Wang, J., Ng, K. K., Chong, J. S. X., Qian, X., Lim, J. K. W., … Zhou, J. H. (2021). Distinct network topology in Alzheimer’s disease and behavioral variant frontotemporal dementia. Alzheimer’s Research & Therapy, 13(1), 13. 10.1186/s13195-020-00752-w, 33407913 PMC7786961

[bib30] Nigro, S., Tafuri, B., Urso, D., De Blasi, R., Cedola, A., Gigli, G., … Frontotemporal Lobar Degeneration Neuroimaging Initiative. (2022). Altered structural brain networks in linguistic variants of frontotemporal dementia. Brain Imaging and Behavior, 16(3), 1113–1122. 10.1007/s11682-021-00560-2, 34755293 PMC9107413

[bib31] Nigro, S., Tafuri, B., Urso, D., De Blasi, R., Frisullo, M. E., Barulli, M. R., … Logroscino, G. (2021). Brain structural covariance networks in behavioral variant of frontotemporal dementia. Brain Sciences, 11(2), 192. 10.3390/brainsci11020192, 33557411 PMC7915789

[bib32] Petersen, R. C. (2004). Mild cognitive impairment as a diagnostic entity. Journal of Internal Medicine, 256(3), 183–194. 10.1111/j.1365-2796.2004.01388.x, 15324362

[bib33] Rascovsky, K., Hodges, J. R., Knopman, D., Mendez, M. F., Kramer, J. H., Neuhaus, J., … Miller, B. L. (2011). Sensitivity of revised diagnostic criteria for the behavioural variant of frontotemporal dementia. Brain, 134(9), 2456–2477. 10.1093/brain/awr179, 21810890 PMC3170532

[bib34] Ravindranath, V., & Sundarakumar, J. S. (2021). Changing demography and the challenge of dementia in India. Nature Reviews Neurology, 17(12), 747–758. 10.1038/s41582-021-00565-x, 34663985 PMC8522537

[bib35] Reyes, P., Ortega-Merchan, M. P., Rueda, A., Uriza, F., Santamaria-García, H., Rojas-Serrano, N., … Matallana, D. (2018). Functional connectivity changes in behavioral, semantic, and nonfluent variants of frontotemporal dementia. Behavioural Neurology, 2018, 9684129. 10.1155/2018/9684129, 29808100 PMC5902123

[bib36] Rogalski, E., Cobia, D., Harrison, T. M., Wieneke, C., Thompson, C. K., Weintraub, S., & Mesulam, M.-M. (2011). Anatomy of language impairments in primary progressive aphasia. Journal of Neuroscience, 31(9), 3344–3350. 10.1523/JNEUROSCI.5544-10.2011, 21368046 PMC3112000

[bib37] Sanabria-Diaz, G., Melie-García, L., Iturria-Medina, Y., Alemán-Gómez, Y., Hernández-González, G., Valdés-Urrutia, L., … Valdés-Sosa, P. (2010). Surface area and cortical thickness descriptors reveal different attributes of the structural human brain networks. NeuroImage, 50(4), 1497–1510. 10.1016/j.neuroimage.2010.01.028, 20083210

[bib38] Schäfer, T., & Ecker, C. (2020). fsbrain: An R package for the visualization of structural neuroimaging data [Preprint]. bioRxiv. 10.1101/2020.09.18.302935

[bib39] Sedeño, L., Piguet, O., Abrevaya, S., Desmaras, H., García-Cordero, I., Baez, S., … Ibanez, A. (2017). Tackling variability: A multicenter study to provide a gold-standard network approach for frontotemporal dementia. Human Brain Mapping, 38(8), 3804–3822. 10.1002/hbm.23627, 28474365 PMC6867023

[bib40] Seeley, W. W., Crawford, R. K., Zhou, J., Miller, B. L., & Greicius, M. D. (2009). Neurodegenerative diseases target large-scale human brain networks. Neuron, 62(1), 42–52. 10.1016/j.neuron.2009.03.024, 19376066 PMC2691647

[bib41] Tao, Y., Ficek, B., Rapp, B., & Tsapkini, K. (2020). Different patterns of functional network reorganization across the variants of primary progressive aphasia: A graph-theoretic analysis. Neurobiology of Aging, 96, 184–196. 10.1016/j.neurobiolaging.2020.09.007, 33031971 PMC7722221

[bib42] Tao, Y., Ficek, B., Wang, Z., Rapp, B., & Tsapkini, K. (2021). Selective functional network changes following tDCS-augmented language treatment in primary progressive aphasia. Frontiers in Aging Neuroscience, 13, 681043. 10.3389/fnagi.2021.681043, 34322010 PMC8311858

[bib43] Thibaut, A., Panda, R., Annen, J., Sanz, L. R. D., Naccache, L., Martial, C., … Gosseries, O. (2021). Preservation of brain activity in unresponsive patients identifies MCS star. Annals of Neurology, 90(1), 89–100. 10.1002/ana.26095, 33938027 PMC8252577

[bib44] Vijverberg, E. G. B., Tijms, B. M., Dopp, J., Hong, Y. J., Teunissen, C. E., Barkhof, F., … Pijnenburg, Y. A. L. (2017). Gray matter network differences between behavioral variant frontotemporal dementia and Alzheimer’s disease. Neurobiology of Aging, 50, 77–86. 10.1016/j.neurobiolaging.2016.11.005, 27940352

[bib45] Wang, X., Cui, X., Ding, C., Li, D., Cheng, C., Wang, B., & Xiang, J. (2021). Deficit of cross-frequency integration in mild cognitive impairment and Alzheimer’s disease: A multilayer network approach. Journal of Magnetic Resonance Imaging, 53(5), 1387–1398. 10.1002/jmri.27453, 33244827 PMC8247269

[bib46] Warren, J. D., Rohrer, J. D., & Rossor, M. N. (2013). Frontotemporal dementia. BMJ, 347, f4827. 10.1136/bmj.f4827, 23920254 PMC3735339

