# Supplementary material for: Multiplex connectomics reveal altered networks in frontotemporal dementia: A multisite study
Source: Netw Neurosci. 2025 Apr 30;9(2):615–30. doi: 10.1162/netn_a_00448 (PMC12140567; doi:10.1162/netn_a_00448)
Supplement: Supplementary file 1 [file netn-9-2-615-s001.pdf]

Title: Multiplex connectomics reveal altered networks in frontotemporal dementia: A multisite study

Sunil Kumar Khokhar[1], Manoj Kumar[1], Faheem Arshad[2], Sheetal Goyal[2], Megha Tiwari[2], Nithin Thanissery[2], Subasree Ramakrishnan[2], Chandana Nagaraj[1], Rajan Kashyap[1], Sandhya Mangalore[1], Tapan K Gandhi[3], Suvarna Alladi[2], Rose Dawn Bharath[1], for the frontotemporal lobar degeneration neuroimaging initiative*

Authors affiliations

1. Department of Neuroimaging and Interventional Radiology, NIMHANS, Bengaluru
2. Department of Neurology, NIMHANS, Bengaluru
3. Department of Electrical Engineering, IIT Delhi

* Data used in preparation of this article were obtained from the Frontotemporal Lobar Degeneration Neuroimaging Initiative (FTLDNI) database. The investigators at NIFD/FTLDNI contributed to the design and implementation of FTLDNI and/or provided data, but did not participate in analysis or writing of this report. Lists of authors and their affiliations appear at the end of the paper.

Supplementary Table 1. The parameters of acquisition of MPRAGE sequence and FDG PET for Site 1 and 2.

**MRI**	**Scanner**	**Sequence**	**TR (ms)**	**TE (ms)**	**FA**	**FOV**	**No of Slices**	**Slice thickness**	**Matrix & Voxel size**
Site1	Biograph mMR, Siemens(3T)	MPRAGE	2300	2.42	9°	256X256	192	1 mm	256×256× 192 (1×1×1)
Site2	Discovery MR 750, GE	MPRAGE	7360	3.04	8°	260X260	166	1.2 mm	260×260× 166 (1×1×1.2)
**PET**	**Scanner**	**PET Tracer**	**Dose**	**Acquisition Time**	**FDG tracer**				**Matrix & Voxel size**
Site1	Biograph mMR, Siemens	FDG	185 MBq (5.0 mCi) ± 10%	15 min	acquisition at 30 min post-injection of FDG tracer.				344×344×127 (1.04×1.04×2.03)
Site2	Discovery RX, GE	FDG	185 MBq (5.0 mCi) ± 10%	30 min (6X5 mi frames) we used 15 min (3x5)	acquisition at 30 min post-injection of FDG tracer.				256×256×47 (2×2×3.27)

Supplementary Results

1. Multiplex global measures

1.1 Overlapping degree average (Global):

When comparing PPA to CN, bvFTD to MCI, bvFTD to CN, PPA to MCI, and bvFTD to PPA (Site 1 & 2) no significant differences were observed in the Overlapping Degree.

1.2 Multiplex participation coefficient (Global):

There was an increase in the global MPC in patients with PPA when compared to MCI in Site 1 (d=0.4, 0.45, 0.5, 0.55, 0.6, 0.65, 0.7, diff= 0.24, 0.31, 0.37, 0.44, 0.46, 0.44, 0.44). Increased global MPC was also seen in bvFTD and PPA when compared with CN (d=0.4, 0.45, 0.5, 0.55, 0.6, 0.65, 0.7; diff= 0.32, 0.4, 0.45, 0.49, 0.48, 0.45, 0.36 and d=0.55, 0.6, 0.65, 0.7; diff= 0.25, 0.27, 0.26, 0.18 respectively) and when bvFTD was compared with PPA in site 2 (d= 0.4, 0.45, 0.5; diff= -0.2, -0.23, -0.24). When comparing bvFTD to MCI, and bvFTD to PPA in site 1, no significant differences were observed in the global MPC.

2. Multiplex Participation coefficient (Nodal)

bvFTD vs MCI/CN

Following regions showed the significant increase in MPC nodal measures in bvFTD in compare to CN and MCI (Supplementary Figure 1-A&B).

bvFTD>CN		bvFTD > MCI	
**Left**	**Right**	**Left**	**Right**
G_and_S_cingulMidPost	G_and_S_cingulMidAnt	G_cingulPostventral	G_cingulPostventral
G_and_S_frontomargin	G_and_S_cingulMidPost	G_subcallosal	G_subcallosal
G_front_infOpercular	G_and_S_paracentral	S_collat_transv_ant	S_circular_insula_sup
G_front_infTriangul	G_front_infOpercular		S_orbital_medolfact
G_front_middle	G_front_sup		S_suborbital
G_front_sup	G_insular_short		S_temporal_inf
G_Ins_lg_and_S_cent_ins	G_occipital_sup		S_temporal_transverse
G_occipital_sup	G_parietal_sup		
G_parietal_sup	G_precuneus		
G_temp_supG_T_transv	G_temp_supG_T_transv		

Lat_FisantHorizont	G_temp_supPlan_tempo		
Lat_FisantVertical	Lat_FisantHorizont		
S_circular_insula_ant	S_cingulMarginalis		
S_circular_insula_sup	S_circular_insula_ant		
S_front_inf	S_circular_insula_inf		
S_front_middle	S_front_sup		
S_front_sup	S_orbitalH_Shaped		
S_orbital_lateral	S_pericallosal		
S_orbital_medolfact	S_suborbital		
S_orbitalH_Shaped	S_subparietal		
S_precentralinfpart	S_temporal_transverse		
S_suborbital			

PPA vs MCI/CN

Following regions showed the significant differences in MPC nodal measures in PPA in compare to CN and MCI (Supplementary Figure 1-C&D).

**PPA>CN**		**PPA > MCI**	
**Left**	**Right**	**Left**	**Right**
G_and_S_transv_frontopol	G_and_S_frontomargin **(CN>PPA)**	G_and_S_cingulAnt	G_and_S_cingulAnt
G_cingulPostventral	G_and_S_occipital_inf	G_and_S_cingulMidPost	G_front_infOrbital
G_front_middle	G_cingulPostventral	G_front_middle	G_Ins_lg_and_S_cent_ins
G_front_sup	G_cuneus	G_front_sup	G_temp_supG_T_transv
G_insular_short	G_temp_supLateral	S_circular_insula_ant	S_cingulMarginalis
G_temp_supG_T_transv	G_temporal_inf	S_circular_insula_sup	S_front_sup
G_temp_supLateral	Lat_FisantHorizont	S_front_middle	
G_temporal_inf	Lat_FisantVertical	S_orbital_lateral	
G_temporal_middle	Pole_temporal		
Lat_FisantHorizont	S_circular_insula_inf		
Lat_FisantVertical	S_collat_transv_ant		
Pole_temporal	S_collat_transv_post **(CN>PPA)**		
S_circular_insula_ant	S_oc_middle_and_Lunatus		
S_circular_insula_inf	S_orbital_lateral		
S_collat_transv_ant	S_pericallosal		

S_collat_transv_post<br>**(CN>PPA)**	S_suborbital		
S_front_sup	S_temporal_transverse		
S_interm_primJensen			
S_octemp_lat			
S_orbital_medolfact			
S_pericallosal			
S_precentralinfpart			
S_temporal_inf			
S_temporal_sup			

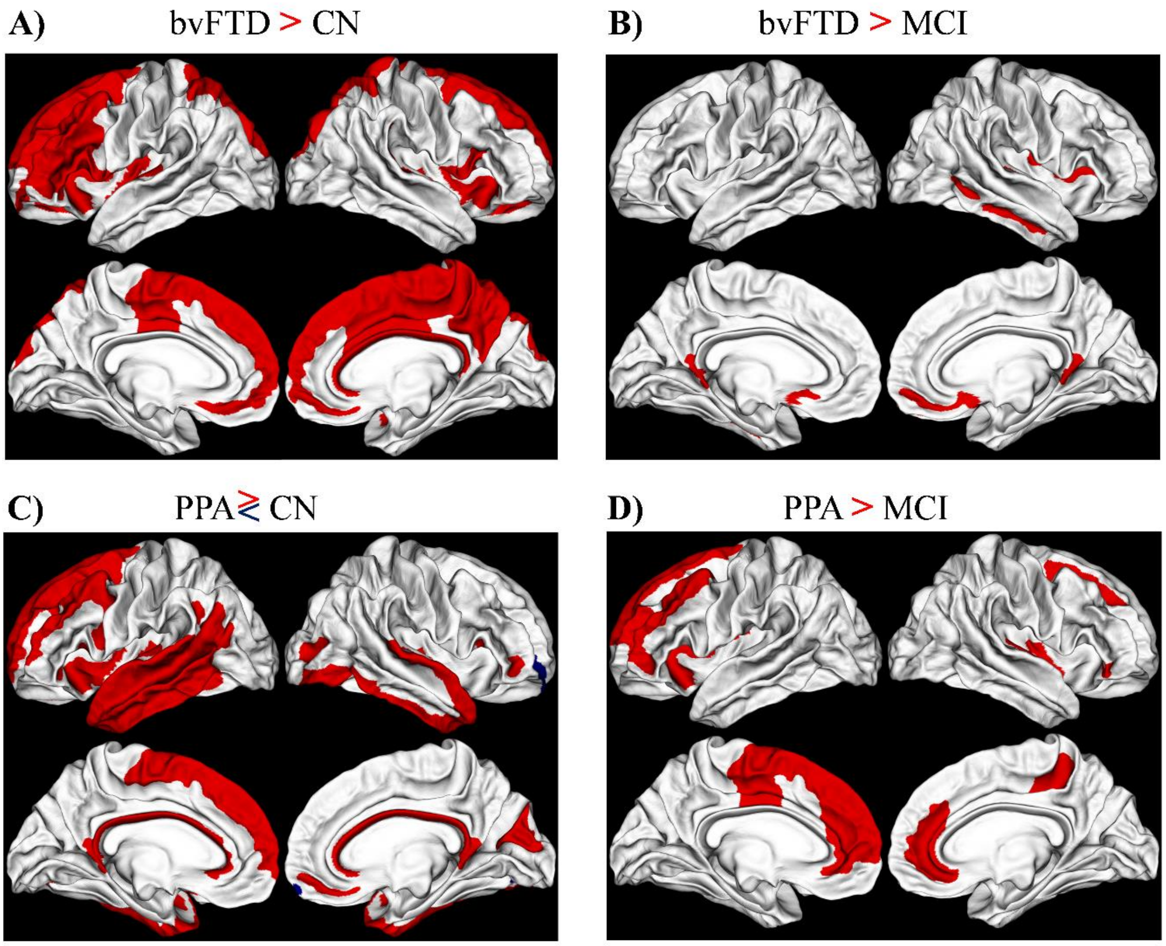

Supplementary Figure 1: Nodal multiplex participation coefficient shows the significant differences between the group bvFTD and CN (A), bvFTD and MCI (B), PPA and CN (C), and PPA and MCI(D). (FDR corrected at $p< 0.05$)

bvFTD vs PPA:

Following regions showed the significant difference in MPC nodal measures in PPA in compare to bvFTD (Supplementary Figure 2-A&B).

**bvFTD vs PPA (site 1)**			
**Left**		**Right**	
G_cingulPostventral	PPA>bvFTD	G_cingulPostventral	PPA>bvFTD
G_cuneus	bvFTD>PPA	S_collat_transv_ant	bvFTD>PPA
G_front_infTriangul	bvFTD>PPA	S_orbital_medolfact	PPA>bvFTD
G_octemp_medLingual	bvFTD>PPA		
G_octemp_medParahip	PPA>bvFTD		
G_precentral	bvFTD>PPA		
S_circular_insula_sup	bvFTD>PPA		
S_collat_transv_ant	PPA>bvFTD		
S_postcentral	bvFTD>PPA		
**bvFTD vs PPA (site 2)**			
**Left**		**Right**	
G_and_S_cingulMidAnt	bvFTD>PPA	G_insular_short	PPA> bvFTD
G_temp_supG_T_transv	PPA> bvFTD	G_precuneus	PPA> bvFTD
Lat_FisantHorizont	PPA> bvFTD	G_temp_supG_T_transv	PPA> bvFTD
S_interm_primJensen	bvFTD>PPA	G_temp_supPlan_tempo	PPA> bvFTD
S_octemp_lat	bvFTD>PPA	Lat_FisantHorizont	PPA> bvFTD
S_orbital_medolfact	PPA> bvFTD	Lat_FisantVertical	bvFTD>PPA
S_orbitalH_Shaped	PPA> bvFTD	S_calcarine	bvFTD>PPA
		S_collat_transv_ant	bvFTD>PPA
		S_suborbital	bvFTD>PPA
		S_temporal_transverse	PPA> bvFTD

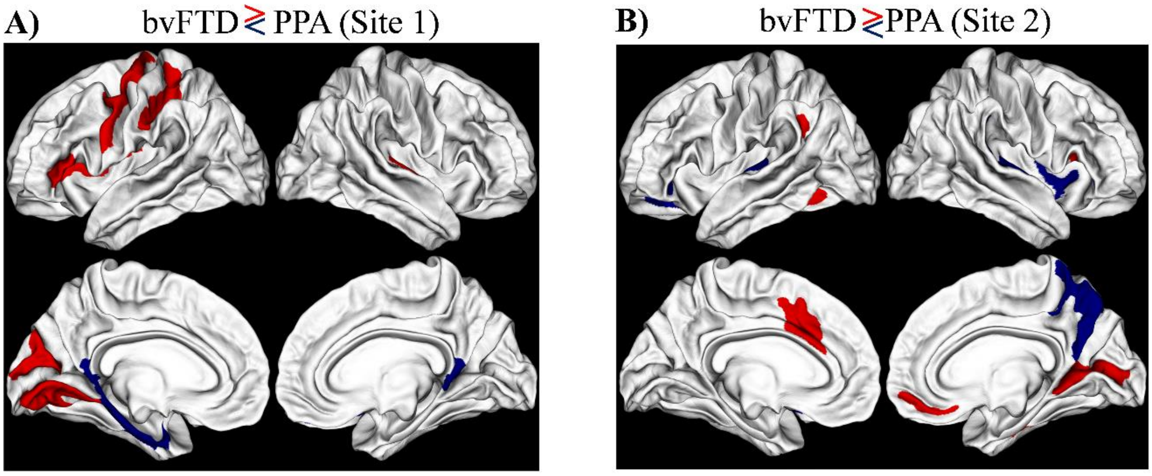

Supplementary Figure 2: Nodal multiplex participation coefficient significant differences between the bvFTD and PPA from site1 (A) and bvFTD vs PPA from site 2 (B). (FDR corrected at $p < 0.05$)

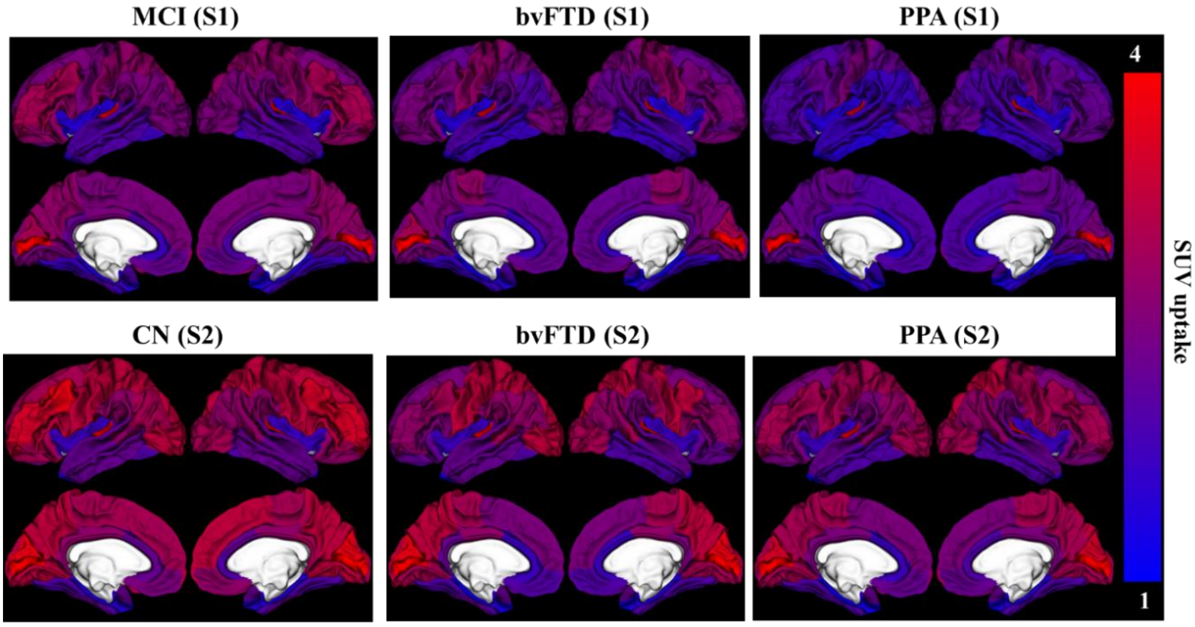

Supplementary Figure 3*: Mean SUV image for FDG uptake of each group in site-1 and site-2.*

Supplementary Table 2. FDG mean SUV+standard deviation values for each group.

	Site-1						Site-2					
ROI	bvFTD		PPA		MCI		bvFTD		PPA		CN	
	Mean SUV	STD	Mean SUV	STD	Mean SUV	STD	Mean SUV	STD	Mean SUV	STD	Mean SUV	STD
lh_isthmuscingulate	1.98	0.49	1.85	0.33	2.14	0.41	2.18	0.30	2.24	0.34	2.59	0.33
rh_isthmuscingulate	2.10	0.46	1.95	0.50	2.32	0.44	2.31	0.32	2.39	0.41	2.68	0.36
lh_medialorbitofrontal	2.00	0.31	1.82	0.24	2.13	0.36	1.92	0.43	2.04	0.34	2.39	0.35
rh_medialorbitofrontal	2.02	0.37	1.93	0.28	2.19	0.38	1.91	0.47	2.13	0.34	2.47	0.31
lh_posteriorcingulate	2.09	0.33	1.84	0.24	2.23	0.41	2.42	0.36	2.32	0.32	2.52	0.29
rh_posteriorcingulate	2.12	0.34	2.03	0.26	2.28	0.39	2.48	0.34	2.37	0.34	2.55	0.29
lh_precuneus	2.32	0.48	2.05	0.33	2.48	0.43	2.80	0.46	2.69	0.34	2.85	0.32
rh_precuneus	2.30	0.45	2.11	0.33	2.41	0.39	2.82	0.41	2.71	0.38	2.80	0.31
lh_rostralanteriorcingulate	1.63	0.21	1.50	0.25	1.77	0.32	1.69	0.30	1.73	0.34	2.01	0.25
rh_rostralanteriorcingulate	1.65	0.24	1.54	0.26	1.80	0.34	1.65	0.46	1.81	0.40	1.98	0.25
lh_lateralorbitofrontal	2.24	0.42	1.97	0.31	2.44	0.37	2.13	0.42	2.30	0.31	2.60	0.32
rh_lateralorbitofrontal	2.21	0.40	2.13	0.36	2.44	0.36	2.09	0.50	2.31	0.34	2.58	0.27
lh_parahippocampal	1.38	0.28	1.35	0.20	1.41	0.22	1.39	0.21	1.32	0.26	1.47	0.15
rh_parahippocampal	1.39	0.31	1.34	0.27	1.40	0.20	1.46	0.20	1.44	0.23	1.53	0.15
lh_caudalanteriorcingulate	1.68	0.26	1.57	0.28	1.90	0.35	1.66	0.66	1.70	0.57	2.09	0.24
rh_caudalanteriorcingulate	1.81	0.24	1.71	0.26	2.00	0.41	1.72	0.54	1.83	0.47	2.18	0.29
lh_inferiortemporal	1.72	0.36	1.57	0.33	1.86	0.28	2.01	0.28	1.94	0.27	2.14	0.37
rh_inferiortemporal	1.71	0.34	1.66	0.34	1.81	0.27	1.94	0.28	1.91	0.28	2.02	0.31
lh_middletemporal	1.78	0.43	1.62	0.38	1.91	0.32	2.02	0.27	1.95	0.28	2.14	0.37
rh_middletemporal	1.79	0.48	1.76	0.35	1.96	0.29	1.97	0.30	1.96	0.30	2.13	0.33
lh_parsopercularis	2.19	0.57	1.87	0.41	2.52	0.43	2.38	0.51	2.36	0.48	2.90	0.35
rh_parsopercularis	2.16	0.53	1.99	0.40	2.53	0.40	2.49	0.48	2.47	0.45	2.89	0.32
lh_parsorbitalis	2.23	0.59	1.93	0.31	2.50	0.43	2.41	0.59	2.43	0.43	2.67	0.37
rh_parsorbitalis	2.17	0.63	2.09	0.35	2.42	0.38	2.18	0.58	2.43	0.35	2.68	0.33
lh_parstriangularis	2.26	0.73	1.87	0.37	2.62	0.46	2.33	0.56	2.36	0.43	2.90	0.42
rh_parstriangularis	2.22	0.65	2.11	0.38	2.61	0.41	2.31	0.58	2.47	0.43	2.91	0.39
lh_insula	1.53	0.22	1.43	0.27	1.54	0.18	1.67	0.18	1.63	0.19	1.72	0.20
rh_insula	1.53	0.22	1.44	0.20	1.55	0.17	1.71	0.29	1.62	0.18	1.72	0.18
lh_rostralmiddlefrontal	2.33	0.80	1.99	0.36	2.64	0.46	2.43	0.48	2.68	0.45	3.01	0.40
rh_rostralmiddlefrontal	2.31	0.76	2.13	0.41	2.65	0.50	2.44	0.54	2.73	0.46	3.07	0.36
lh_supramarginal	2.07	0.62	1.77	0.36	2.23	0.34	2.41	0.29	2.32	0.32	2.56	0.36
rh_supramarginal	2.05	0.64	1.86	0.30	2.23	0.30	2.36	0.38	2.34	0.33	2.48	0.31
lh_caudalmiddlefrontal	2.34	0.81	2.01	0.42	2.60	0.53	2.67	0.62	2.61	0.57	3.22	0.40
rh_caudalmiddlefrontal	2.35	0.80	2.08	0.40	2.63	0.45	2.88	0.56	2.76	0.57	3.19	0.37
lh_superiortemporal	1.86	0.43	1.73	0.34	1.84	0.27	2.14	0.28	2.12	0.28	2.10	0.29
rh_superiortemporal	1.87	0.44	1.84	0.34	1.90	0.26	2.18	0.30	2.10	0.27	2.18	0.25
lh_cuneus	2.65	0.58	2.40	0.39	2.60	0.50	3.34	0.77	3.14	0.55	3.30	0.44
rh_cuneus	2.60	0.54	2.31	0.32	2.57	0.50	3.20	0.63	3.03	0.56	3.21	0.49
lh_lateraloccipital	2.22	0.75	2.07	0.41	2.32	0.50	2.80	0.55	2.71	0.55	2.94	0.50
rh_lateraloccipital	2.25	0.71	2.11	0.32	2.30	0.42	2.63	0.50	2.60	0.47	2.74	0.43
lh_fusiform	1.72	0.30	1.66	0.28	1.75	0.24	1.98	0.26	1.95	0.26	1.97	0.21
rh_fusiform	1.70	0.31	1.58	0.30	1.73	0.27	1.93	0.24	1.92	0.27	1.89	0.19
lh_lingual	2.26	0.53	2.15	0.28	2.26	0.37	2.75	0.51	2.69	0.47	2.80	0.38
rh_lingual	2.20	0.49	2.11	0.30	2.19	0.38	2.68	0.53	2.61	0.49	2.80	0.40
lh_bankssts	2.09	0.57	1.84	0.42	2.19	0.39	2.72	0.30	2.41	0.34	2.57	0.38
rh_bankssts	2.13	0.52	1.89	0.42	2.13	0.29	2.68	0.45	2.46	0.33	2.50	0.28
lh_entorhinal	1.52	0.21	1.65	0.43	1.64	0.24	1.40	0.14	1.34	0.28	1.42	0.28
rh_entorhinal	1.53	0.24	1.43	0.31	1.58	0.28	1.40	0.31	1.43	0.27	1.53	0.22
lh_frontalpole	2.18	0.82	1.98	0.55	2.48	0.76	2.06	1.12	2.47	0.71	2.84	0.56
rh_frontalpole	2.29	0.78	2.32	0.34	2.54	0.77	2.02	1.20	2.66	0.56	2.89	0.46
lh_inferiorparietal	1.96	0.52	1.77	0.40	2.23	0.46	2.54	0.37	2.42	0.33	2.66	0.44
rh_inferiorparietal	2.03	0.57	1.91	0.39	2.27	0.34	2.46	0.38	2.43	0.34	2.59	0.35
lh_paracentral	2.47	0.59	2.19	0.37	2.34	0.36	2.72	0.44	2.67	0.31	2.67	0.27
rh_paracentral	2.45	0.52	2.19	0.35	2.34	0.38	2.73	0.50	2.63	0.28	2.64	0.25
lh_pericalcarine	3.72	1.41	3.46	0.75	3.69	0.74	4.32	1.20	4.11	1.00	4.35	0.86
rh_pericalcarine	3.60	1.38	3.37	0.69	3.57	0.69	4.34	1.27	4.08	0.99	4.52	0.92
lh_postcentral	2.57	0.75	2.28	0.36	2.46	0.43	2.83	0.34	2.73	0.34	2.81	0.35
rh_postcentral	2.55	0.78	2.31	0.39	2.45	0.38	2.68	0.40	2.73	0.35	2.84	0.35
lh_precentral	2.45	0.67	2.14	0.29	2.38	0.41	2.82	0.40	2.66	0.34	2.85	0.34
rh_precentral	2.50	0.78	2.16	0.32	2.41	0.39	2.87	0.47	2.69	0.38	2.87	0.32
lh_superiorfrontal	2.12	0.59	1.90	0.30	2.33	0.43	2.21	0.39	2.29	0.41	2.79	0.32
rh_superiorfrontal	2.15	0.60	1.99	0.31	2.34	0.42	2.29	0.38	2.40	0.40	2.80	0.31
lh_superiorparietal	2.33	0.63	2.10	0.48	2.48	0.44	2.85	0.47	2.74	0.38	2.92	0.35
rh_superiorparietal	2.30	0.53	2.15	0.39	2.46	0.44	2.89	0.46	2.80	0.41	2.92	0.35
lh_temporalpole	1.43	0.17	1.32	0.26	1.48	0.22	1.30	0.30	1.25	0.31	1.48	0.25
rh_temporalpole	1.39	0.22	1.27	0.26	1.36	0.21	1.23	0.64	1.21	0.53	1.45	0.20
lh_transversetemporal	3.52	0.91	3.30	0.77	3.61	0.73	3.82	0.71	3.63	0.58	3.79	0.53
rh_transversetemporal	3.80	1.06	3.59	0.69	3.85	0.73	4.01	0.67	3.88	0.80	3.98	0.64
